# Diversity of *Bacteroidaceae* family in gut microbiota of patients with chronic kidney disease and end stage renal disease

**DOI:** 10.34172/hpp.2023.29

**Published:** 2023-09-11

**Authors:** Siamak Amini Khiabani, Setareh Haghighat, Hamid Tayebi Khosroshahi, Mohammad Asgharzadeh, Hossein Samadi Kafil

**Affiliations:** ^1^Department of Microbiology, Faculty of Advanced Science and Technology, Tehran Medical Sciences, Islamic Azad University, Tehran, Iran; ^2^Research Center for Pharmaceutical Nanotechnology, Tabriz University of Medical Sciences, Tabriz, Iran; ^3^Biotechnology Research Center, Tabriz University of Medical Sciences, Tabriz, Iran; ^4^Drug Applied Research Center, Faculty of Medicine, Tabriz University of Medical Sciences, Tabriz, Iran

**Keywords:** Bacteroidaceae, Chronic kidney disease, End-stage renal disease, Next generation sequencing

## Abstract

**Background:**

Human intestine microbiota are known to be directly and indirectly altered during some diseases such as kidney complications. Bacteroides is considered as the main and the most abundant phylum among human gut microbiota, which has been classified as enterotype 1. This study aimed to assess the abundance of Bacteroides spp. in fecal flora of end-stage renal disease (ESRD) and chronic kidney disease (CKD) patients and compare it with the Bacteroides composition among fecal flora of healthy individual.

**Methods:**

Fresh fecal samples were collected from 20 CKD/ESRD patients and 20 healthy individual without any kidney complications. The pure microbial DNA was extracted by QIAamp Stool Mini Kit from stool samples. MiSeq system was used to analyze the intestinal composition by next generation sequencing method.

**Results:**

A number of 651 bacterial strains were isolated and identified from 40 fecal samples of both patients and healthy groups. Bioinformatics analysis defined 18 different types of Bacteroides species which included 2.76% of all strains. Statistical analysis showed no significant difference between study groups (*P*>0.05). In both healthy and patient groups three species including *B. dorei*, *B. uniformis*, and *B. ovatus* have allocated the most abundance to themselves. The lowest abundance was related to *B. eggerthii*, *A. furcosa* and *B. barnesiae* among CKD/ESRD patients and *A. furcosa*, *B. barnesiae*, and *B. coprocola* had the lowest abundance among healthy people.

**Conclusion:**

This study indicates despite all previous evidence of *Bacteroides* role in gut microbiota, it had no different distribution between healthy persons and CKD/ESRD patients.

## Introduction

*Bacteroidaceae* family is belonged to Bacteroidetes class and phylum. The phylum Bacteroidetes can be found in the environment for instance in sea water, soil, and sediments as well as colonized on the skin of animals and into the guts.^1^ Approximately, around 20%-80% of the gut microbiota relates to Bacteroidetes phylum in healthy adults which the genera of Parabacteroides, Bacteroides, Alistipes, and Prevotella are categorized in this phylum.^2,3^*Bacteroidales*order has a significant abundance in the human gut so that every gram of human feces has a high concentration, which reaches up to 10^9^-10^11^ CFU.^4^ The genera Bacteroides has been identified as gram-negative, obligate anaerobes, rod-shaped with round ends, non-motile, and non-spore-forming which has known as one of the major genera of microbiota composition with more than 30 species.^5,6^

 A large variety and myriad microorganisms have been colonized in human intestine which is called microbiota.^7,8^ Two major phyla: the gram-positive Firmicutes and the gram-negative high CG% Bacteroidetes have formed the microbiota population and the other phyla including Fusobacteria, Actinobacteria, and Verrucomicrobia phyla have been categorized at subdominant levels.^5^ Gut microbiota is a vast world that has many beneficial effects on human body, such as helping to food digestion, producing of hormones and essential vitamins like K and B12, modulating and developing of immune system and powerfully forming a natural defense to limit the infections caused by intestinal pathogens.^9,10^ Some studies also presented that the possible changes in species/phyla levels of Bacteroidetes and Firmicutes can be considered as obesity factors in children.^11^ Another study has reported that Bacteroides species could be widely effective in the treatment of intestinal colitis, metabolic disorders, immune dysfunctions, and cancer prevention which Bacteroides genus is considered as a new beneficial probiotic candidates.^12^ In addition, the members of *Bacteroidaceae* family also decreased inflammation response by regulating cytokine expression and lymphocytes.^13^

 Chronic kidney disease (CKD) has known as a key determinant of noncommunicable disease which can progress toward end-stage renal disease (ESRD).^14^ Both developed and developing nations report high numbers of cases with CKD annually that can alter the intestinal microbiota composition and microbial metabolism quantitatively and qualitatively.^15,16^ Intestine microbiota has contributed to the production of important metabolic substances. On the other hand, the uremic solutes such as indoxyl sulfate and p-cresyl sulfate can be generated by gut microbiota.^17^ Patients who suffer from CKD have shown an altered combination of gut microbiota which have been correlated to the dietary interventions and therapeutic condition and the uremic milieu, result in high production of the uremic solutes.^18^ Several studies have reported a statistical association between mortality and circulating levels of the uremic toxins.^18^ In present study, we assessed changes in the abundance and diversity of *Bacteroides* spp. in intestinal flora of CKD and ESRD patients by comparing differences between healthy humans.

## Materials and Methods

###  Sample collection

 The present trial enrolled 20 patients with CKD and ESRD undergoing hemodialysis from the kidney transplantation ward of Imam-Reza teaching hospital, Tabriz, Iran. On the other hand, 20 healthy volunteers were joined to the study as control group. Fresh fecal samples of both case and control groups were directly collected from the anus of individuals and transferred into the sterile containers and were stored at -80 ºC until further processes. Before participating in the investigation, before participating in the research, written informed consent was signed by all patients and healthy group. Our ESRD patients in this investigation had some underlying diseases including chronic pyelonephritis, glomerulonephritis, hypertensive nephrosclerosis, polycystic kidney disease, post renal and urolithiasis, urolithiasis and systemic lupus erythematosus. Exclusion criteria include patients with some complications such as intestinal disease or colectomy, cholecystectomy, and diabetes, also patients suffering infections, inflammatory disorders, autoimmune diseases and patients who had received antibiotics within three months before enrolling in the study.

###  DNA extraction and PCR amplification

 First of all four grams of mixed and homogenized fresh fecal samples were weighted for extraction of pure DNA. Microbial DNA was isolated from the fecal mixture using the QIAamp Stool Mini Kit (QIAGEN, Germany), according to the manufacturer’s instruction.^19^ The Thermo NanoDrop 2000 spectrophotometer (Thermo Scientific, MA, USA) was used to find the exact amount of DNA in each fecal sample.^20^ Two sequence of universal bacterial 16srRNA (V3–V4 hypervariable regions) were used for amplification of template DNA and sequencing. The specific sequences in this trial were as follows^21^:

 Illumina V3:

 5’-TCGTCGGCAGCGTCAGATGTGTATAA GAGACAGCCTACGGGNGGCWGCAG-3’

 Illumina V4:

 5’-TCTCGTGGGCTCGGAGATGTGTATAA GAGACAGGACTACHVGGGTATCTAATCC-3’

 The amplification of the target sequences was performed using a T100TM thermal (Bio-Rad, USA). The polymerase chain reaction (PCR) reactions were performed as follows: 95 °C for 5 minutes, followed by 35 cycles of 95 °C for 1 minute, 55 °C for 45 seconds, and 72 °C for 1 minute, with a final extension of 72 °C for 1 minute. The electrophoresis was run in 1% agarose gel in Tris-boric acid-Ethylenediaminetetraacetic acid (EDTA) buffer to assess the PCR products and the gel was stained with ethidium-bromide to be visible under UV light. MiSeq system (100k 2 x 300 bp paired-end reads) (Illumina, USA) was accomplished the sequencing of PCR products in Omega Bioservices company. Bioinformatics analyses were completed by Illumina’s BaseSpace in parallel with Illumina’s in-house QIIme 2 pipeline.

###  Statistical analysis

 Statistical analysis was assessed using programs including GraphPad PRISM 8 and SPSS 20. Statistical analysis was performed to compare case and control groups using the Mann-Whitney nonparametric test and Welch’s *t* test. *P* values < 0.05 were considered statistically significant.

## Results

**Figure 1:**
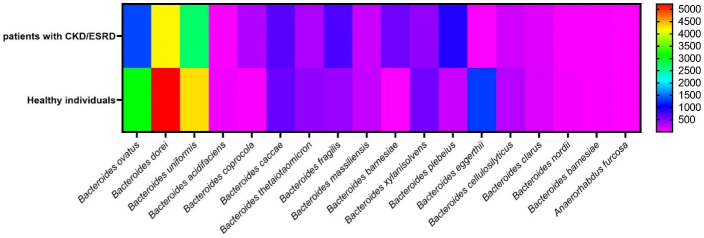
Heatmap graph of the abundance of different species of *Bacteroidaceae* family identified in fecal samples of both patients with CKD/ESRD and healthy individuals. This map indicates number of each species of the family however statistical analysis showed no significant differences (*P* > 0.05)

 A total of 20 patients with CKD/ESRD, 14 patients were male and 6 patients were female with the mean age of 53.20 ± 12.03 years. As well as, a total of 20 healthy individuals, 10 persons were male and 10 were female with the mean age 59.3 ± 7.89 years. The results of MiSeq system demonstrated that 651 bacterial strains were found in 40 fecal samples of both patients and healthy individuals, which 18 strains (596538 reads, 257413 vs. 339125 reads) belong to family *Bacteroidaceae*. The strains were belonged to two genera including *Bacteroides* (17 species) and *Anaerorhabdus* (one species). The most abundance of species in patients with CKD/ESRD were *B. dorei* (32.66%), *B. uniformis* (21.03%) and *B. ovatus* (10.5%) and the lowest were *B. eggerthii* (0.01%), *Anaerorhabdus furcosa* (0.03%) and *B. barnesiae* (0.17%). As well as, the most abundance of species in healthy individuals were *B. dorei* (30.74%), *B. uniformis* (25.38%) and *B. ovatus* (18.3%) and the lowest were *A. furcosa* (0.01%), *B. barnesiae* (0.01%) and *B. coprocola* (0.07%). Using statistical analysis, the abundance of various species did not show any significant difference between the patients and control group (all *P* > 0.05). The abundance of various species is shown in Table 1 and Figure 1.

**Table 1:** The abundance of different species of *Bacteroidaceae* family identified in fecal samples of both patients with CKD/ESRD and healthy individuals

**Species**	**Patient group sum**	**Mean**	**STDEV**	**Min**	**Max**	**Individuals collected**	**Health individual sum**	**Mean**	**STDEV**	**Min**	**Max**	**Individuals collected**	***P*** ** value**
*Bacteroides ovatus*	26630	1331.5	3701.55	4	16463	20	62053	3102.65	8402.18	7	32760	20	0.396
*Bacteroides dorei*	84072	4203.6	8365.95	12	33205	20	104261	5213.05	12075.51	37	50687	20	0.760
*Bacteroides uniformis*	54126	2706.3	6549.13	2	28579	20	86058	4302.9	13938.53	0	62988	19	0.647
*Bacteroides acidifaciens*	554	27.7	116.65	0	523	4	1257	62.85	272.41	0	1220	5	0.600
*Bacteroides coprocola*	6411	320.55	1430.48	0	6398	4	241	12.05	36.83	0	142	3	0.347
*Bacteroides caccae*	13442	672.1	1214.01	0	3728	17	12198	609.9	1369.50	0	4739	15	0.880
*Bacteroides thetaiotaomicron*	6881	344.05	888.37	0	3823	13	8895	444.75	1516.11	0	6774	11	0.799
*Bacteroides fragilis*	14505	725.25	1919.34	0	8016	15	8267	413.35	1476.20	0	6643	13	0.568
*Bacteroides massiliensis*	4287	214.35	703.12	0	2948	10	4609	230.45	947.94	0	4253	9	0.952
*Bacteroides faecis*	11688	584.4	2612.57	0	11684	2	44	2.2	9.84	0	44	1	0.331
*Bacteroides xylanisolvens*	8919	445.95	1395.33	0	6314	15	11422	571.1	1606.37	0	6270	14	0.794
*Bacteroides plebeius*	18231	911.55	2889.86	0	11503	5	4331	216.55	653.14	0	2236	6	0.306
*Bacteroides eggerthii*	27	1.35	4.38	0	19	3	25678	1283.9	5712.37	0	25553	9	0.328
*Bacteroides cellulosilyticus*	3882	194.1	581.03	0	2460	9	5844	292.2	685.65	0	2394	14	0.628
*Bacteroides clarus*	2560	128	527.86	0	2369	10	2684	134.2	421.53	0	1848	10	0.967
*Bacteroides nordii*	672	33.6	87.98	0	371	4	704	35.2	107.60	0	458	6	0.959
*Bacteroides barnesiae*	442	22.1	97.90	0	438	2	540	27	120.75	0	540	1	0.889
*Anaerorhabdus furcosa*	84	4.2	14.81	0	66	3	39	1.95	8.72	0	39	1	0.562

STDEV, standard deviation; Min, minimum; Max, maximum.

## Discussion

 In both CKD patients and healthy individuals, *Bacteroidetes* (~40%), *Firmicutes* (~40%) and *Proteobacteria* (~10%) counted as the predominant phyla in gut microbiota composition.^22^ According to Faith et al^23^ report, during lifetime and human generations, *Bacteroidetes* phyla was more stable in comparison with the phyla *Firmicutes*. Other study has specified Bacteroidetes as the most plenteous phylum were accounted around 41% in both healthy clients and patients.^24^ In present study, among both CKD/ESRD patients and healthy volunteers, 18 strains of a total 651 bacterial strains were related to *Bacteroidaceae* family. On the other hand, the novelty of this study was to compare the abundance and diversity of *Bacteroides* species between fecal samples of CKD/ESRD patients and control group. Consequently, Statistical analysis calculations proved that there was no significant difference in variety of *Bacteroides* species between patients with CKD/ESRD and healthy individuals.

 Numerous studies have examined Bacteroides at different levels of phylum, family, genus and species. Gut microbiota balance changed qualitatively and quantitatively through CKD patients who this imbalance is accompanied with decrease in *Bacteroidaceae*, some *Prevotellaceae*, and particular *Bifidobacterium* and *Lactobacillus*, and increase in the count of *Enterobacteriaceae*, *Lachnospiraceae*, and certain *Ruminococcaceae.*^22,25^ Jiang et al demonstrated that a significant reduction of total bacteria quantity was shown in ESRD patients. Bacteroides was prevalent in ESRD patients whereas Prevotella was enriched in healthy individuals. Also, among ESRD patients, the count of the butyrate producing bacteria such as *Faecalibacterium*, *Roseburia*, *Coprococcus*, *Prevotella*, and *Clostridium* were declined.^22^ Crespo-Salgado et al^26^ found that the gut microbiota composition in pediatric patients with hemodialysis was different compared with healthy controls. Unlike healthy individual, a decrease was shown in *Proteobacteria* members while *Bacteroidetes* was considerably increased in hemodialysis (HD) patients.^26^ In addition to phylum Bacteroidetes, this raises in HD patients, phylum Firmicutes decreases in ESRD patients undergoing peritoneal dialysis (PD).^25^

 In other study about kidney stone disease (KSD), scientists found that gut microbiome can have an essential role in kidney stone formation.^27^ A unique gut microbiota was shown in patients who suffer from nephrolithiasis compared with healthy clients.^27^ Among kidney stone formers, Bacteroides spp. was more prevalent while healthy controls significantly had higher *Prevotella* spp. in microbiota composition.^27^ In a comparison of *Bacteroides* count among KSD and healthy group, *Bacteroidetes* was 3.4 times more plenty in KSD patients.^27^ Li et al assessed patients with both CKD and high systolic blood pressure and observed altered bacterial composition and a reduction in bacterial abundance. Their achievement demonstrated which in hypertension models, the richness of the intestinal *Bacteroidetes* and *Firmicutes* was associated with increased blood pressure.^28^

 Total quantity of fecal microbiota was decreased in ESRD patients unlike healthy controls. Human intestinal microbiota has 3 main enterotype including *Bacteroides* categorized as enterotype 1, *Prevotella* as enterotype 2, and *Ruminococcus* as enterotype 3.^29^ A research study showed that from healthy individual through patients with ESRD, the mentioned enterotype shift from enterotype 2 (Prevotella) to enterotype 1 (Bacteroides), especially *Bacteroidaceae* which have the ability to produce p-cresol increase in ESRD patients.^27^ On the other hand, those bacteria that tend to produce short chain fatty acids like butyrate were declined among patients with ESRD.^27^

 Several studies demonstrated that Bacteroides increase greatly in a variety of diseases, for instance Bacteroides in genus level has increased significantly in diabetes mellitus group.^30^ In addition, the segmented filamentous bacteria specially colonization by *Bacteroidetes* can induce the intestinal infiltration of pro-inflammatory TH17 cells, which is necessary to balance TH1 and TH2 responses.^31^

 In present research, Bacteroides in genus and species level were assessed among both CKD/ESRD patients and healthy people which *B. dorei*, *B. uniformis*, and *B. ovatus* had the highest abundant among both group without any difference. Boente et al^32^ have evaluated the members of *Bacteroidaceae* among other disease like hypertension that some species of Bacteroides including *B. eggerthii*, *B. cellulosilyticus*, and 3 unclassified Bacteroides had important function in patient with hypertension and other Bacteroides spp. such as *B. dorei*, *B. nordii*, and *B. uniformis* have enriched in control group. These findings refer to that the composition of *Bacteroidaceae* members alter not only among CKD/ESRD patients, but also in some other disorders.

## Conclusion

 In summary, several previous knowledge have demonstrated the correlation between increased abundance of Bacteroides members and some disease particularly Kidney problems. Therefore, our findings around comparison of Bacteroides species abundance with CKD/ESRD patients and healthy individuals can extend the previous findings, which there was no significant difference in distribution of *Bacteroidaceae*members among both assessed patients and healthy groups.

 The limitation of our research is that due to financial limitations, it was not possible to conduct research on a larger number of individuals.

## Acknowledgements

 We thank all support for colleagues specially Dr. Pourya Gholizadeh for helps in manuscript preparation and comments.

## Competing Interests

 None to declare.

## Ethical Approval

 This study was conducted based on the confirmation of Medical Ethics Board of Trustees with reference number IR.IAU.PS.REC.1400.483. The result of the Medical Ethics Board of Trustees report is available online (https://ethics.research.ac.ir/EthicsProposalViewEn.php?id = 247843)

## Funding

 This study was done as a dissertation for the Ph.D. degree of Dr. Siamak Amini Khabani and was supported by self-grant and Tabriz University of Medical Sciences and Islamic Azad University. This study was approved in Islamic Azad University with reference number 162314446.
